# Hsa_circ_0032131 knockdown inhibits osteoarthritis progression via the miR-502-5p/PRDX3 axis

**DOI:** 10.18632/aging.203073

**Published:** 2021-05-25

**Authors:** Jin Xu, Xinlong Ma

**Affiliations:** 1 Department of Pain Treatment, Tianjin Hospital, Tianjin 300211, China

**Keywords:** circRNA, OA

## Abstract

Osteoarthritis (OA) is a chronic disease characterized by progressive loss of cartilage and failure of the diarthrodial joint. Circular RNAs (circRNAs) are known to participate in the pathogenesis of multiple diseases, including OA. We investigated the functions of hsa_circ_0032131, a circRNA upregulated in OA, using CHON-001 cells and an *in vivo* OA rat model. CHON-001 cells were treated with interleukin (IL)-1β to mimic OA *in vitro.* IL-1β-induced inhibition of CHON-001 growth was reversed by silencing hsa_circ_0032131. In addition, hsa_circ_0032131 knockdown reversed IL-1β-induced activation of Trx1, Cyclin D and PRDX3, whereas overexpression of *PRDX3*, a direct target of miR-502-5p, reversed this effect. Hsa_circ_0032131 served as a competing endogenous RNA for miR-502-5p. Moreover, knockdown of hsa_circ_0032131 attenuated OA symptoms *in vivo* by inactivating the STAT3 signaling pathway. Thus, silencing of hsa_circ_0032131 inhibited the progression of OA by inactivating the miR-502-5p/PRDX3/Trx1/STAT3 axis, which highlights its potential as a therapeutic target for OA.

## INTRODUCTION

Osteoarthritis (OA) results from progressive loss of joint cartilage that causes chronic pain and disability. It is known to affect approximately 50,000,000 adults in China [1–3]. Although the mainstay of OA treatment includes drugs to relieve the pain and surgery in advanced cases, the associated increase in annual hospitalization and ambulatory care visits results in a high socioeconomic burden [4].

Circular RNAs (circRNAs) are endogenous non-coding RNAs with a stable closed-loop structure that regulate several functions (protein synthesis, post-transcriptional modifications and others) [5, 6]. In addition, circRNAs have been implicated in chondrocyte growth and inflammation in OA [7, 8]. For example, Shen et al. indicated that overexpression of circSERPINE2 could alleviate the apoptosis in the OA cartilage tissues via sponging miR-1271 [9]. Zhou et al. found that circRNA.33186 could aggravate OA symptoms by sponging miR-127-5p [10]. In addition, hsa_circ_0032131 has been reported to be upregulated in OA [11]. Nevertheless, the role of hsa_circ_0032131 in OA remains largely unknown.

MiRNAs are small RNAs of approximately 20 to 25 nucleotides in length that regulate various biological processes by inhibiting the target mRNAs [12]. CircRNAs participate in development of OA in chondrocytes by directly binding to miRNAs to inhibit the translation of target mRNAs—a process known as sponging [9, 13]. For example, circRNA CDR1 promoted OA progression by serving as a sponge for miR-641 [14]. Furthermore, miR-502-5p protected chondrocyte injury against IL-1β by inhibiting *TRAF2*, a critical signaling molecule of the NF-κB signaling [15]. This study investigated the function of hsa_circ_0032131 in OA pathogenesis and the correlation between hsa_circ_0032131 and miR-502-5p.

## RESULTS

### *In vitro* model of OA was successfully established

**Figure 1:**
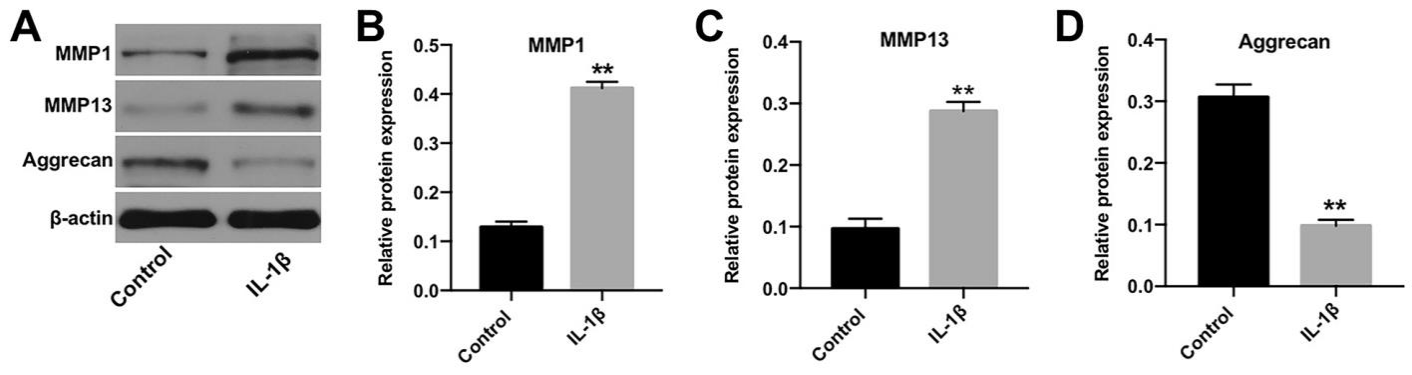
***In vitro* model of OA was successfully established.** (**A**) CHON-001 cells were treated with 10 ng/mL IL-1β for 48 h. Next, the expression of MMP-1, MMP-13, and Aggrecan in CHON-001 cells was detected by western blotting. (**B**) The relative expression of MMP-1 was quantified by normalizing it to that of β-actin. (**C**) The relative expression of MMP-13 was quantified by normalizing it to that of β-actin. (**D**) The relative expression of Aggrecan was quantified by normalizing it to that of β-actin. ^**^p < 0.01 compared with the control.

IL-1β plays a crucial role during the pathogenesis of OA [16]. Jiang et al. found that IL-1β significantly induced the apoptosis, inflammatory response and cartilage matrix destruction of chondrocytes [17]. Thus, to construct *in vitro* model of OA, CHON-001 cells were treated with IL-1β [18]. Western blotting was applied to detect the expression of protein biomarkers in OA [19, 20]. As indicated in Figure 1A, 1C, IL-1β induced the level of MMP-1 and MMP-13, whereas it inactivated Aggrecan in cells (Figure 1A, 1D). These data confirmed that the *in vitro* model of OA was successfully constructed.

### Hsa_circ_0032131silencing rescued the anti-proliferative effect of IL-1β

**Figure 2:**
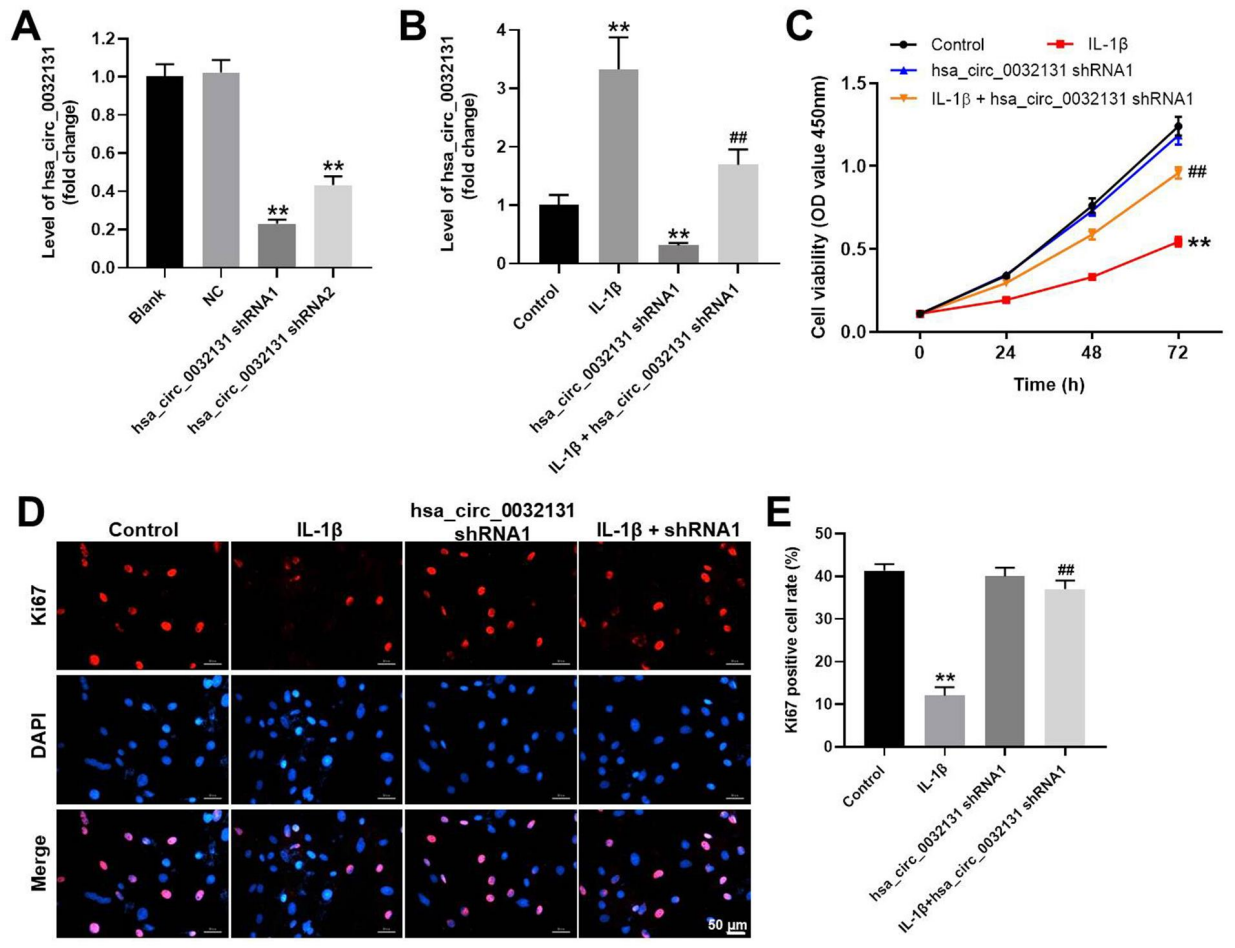
**Knockdown of hsa_circ_0032131 reversed the anti-proliferative effect of IL-1β on CHON-001 cells.** (**A**) CHON-001 cells were transfected with hsa_circ_0032131 shRNA1 or hsa_circ_0032131 shRNA2 for 48 h. Afterward, the expression of hsa_circ_0032131 in CHON-001 cells was detected by RT-qPCR. (**B**) CHON-001 cells were treated with IL-1β, hsa_circ_0032131 shRNA1, or IL-1β + hsa_circ_0032131 shRNA1. Next, the expression of hsa_circ_0032131 in CHON-001 cells was detected by RT-qPCR. (**C**) Cell viability was tested by CCK-8 assay. (**D**) The proliferation of CHON-001 cells was measured by Ki67 staining (red fluorescence). Blue fluorescence indicates DAPI. (**E**) The number of Ki67-positive cells was calculated. ^**^p < 0.01 compared with the control. ^##^*p* < 0.01 compared with IL-1β.

CHON-001 cells were transfected with shRNAs against hsa_circ_0032131. CHON-001 cells were more susceptible to hsa_circ_0032131 shRNA1 than hsa_circ_0032131 shRNA2 (Figure 2A). Therefore, hsa_circ_0032131 shRNA1 was selected for subsequent experiments. The expression of hsa_circ_0032131 in CHON-001 cells was upregulated by IL-1β, whereas this effect was partially reversed after hsa_circ_0032131 knockdown (Figure 2B). In addition, IL-1β decreased CHON-001 cell viability, whereas this effect was partially rescued by hsa_circ_0032131 shRNA1 (Figure 2C). Ki67 cell proliferation assay further confirmed that hsa_circ_0032131 silencing restored IL-1β-induced suppression of cell proliferation (Figure 2D, 2E). To sum up, the knockdown of hsa_circ_0032131 reversed the anti-proliferative effect of IL-1β on CHON-001 cells.

### IL-1β-induced CHON-001 cell apoptosis was reversed by hsa_circ_0032131 knockdown

**Figure 3:**
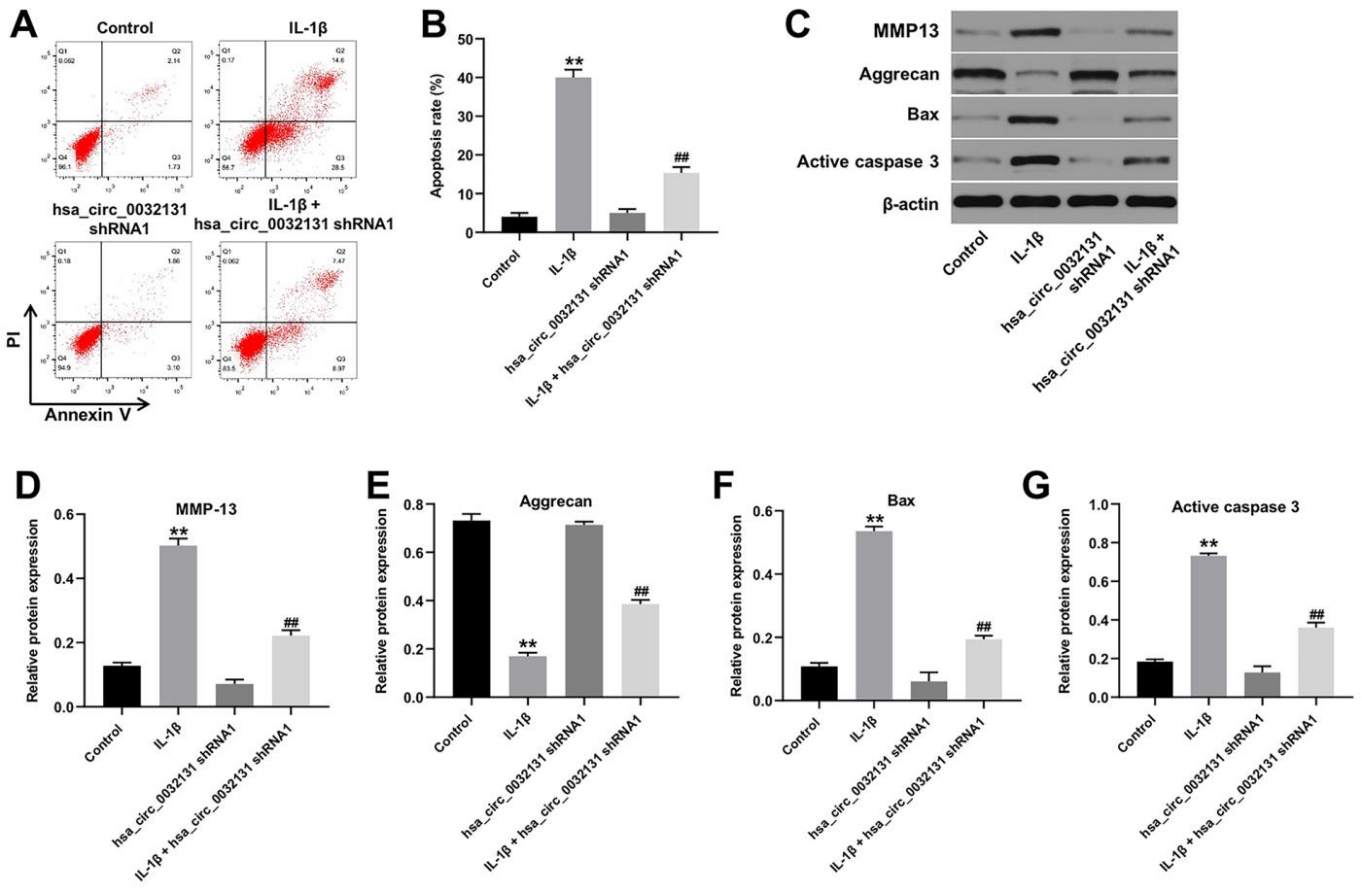
**IL-1β-induced CHON-001 growth inhibition was reversed by hsa_circ_0032131 knockdown.** (**A**, **B**) Apoptosis of CHON-001 cells was measured by flow cytometry. (**C**) The expression of Aggrecan, MMP-13, Bax, and active caspase 3 in CHON-001 cells was detected by western blotting. (**D**) The relative expression of MMP-13 was quantified by normalizing it to that of β-actin. (**E**) The relative expression of Bax was quantified by normalizing it to that of β-actin. (**F**) The relative expression of active caspase 3 was quantified by normalizing it to that of β-actin. (**G**) The relative expression of Aggrecan was quantified by normalizing it to that of β-actin. ^**^p < 0.01 compared with the control. ^##^*p* < 0.01 compared with IL-1β.

Flow cytometry data, shown in Figure 3A, 3B, revealed that IL-1β-induced CHON-001 cell apoptosis was rescued by hsa_circ_0032131 silencing. The expression of MMP-13, Bax, and active caspase 3 was upregulated by IL-1β in CHON-001 cells; this effect was partially rescued by hsa_circ_0032131 shRNA1 (Figure 3C–3F). In contrast, IL-1β-induced decrease in Aggrecan expression was partially reversed by knockdown of hsa_circ_0032131 (Figure 3C, 3G). Altogether, IL-1β-induced CHON-001 cell apoptosis was rescued by knockdown of hsa_circ_0032131.

### Hsa_circ_0032131 sponged miR-502-5p in CHON-001 cells

**Figure 4:**
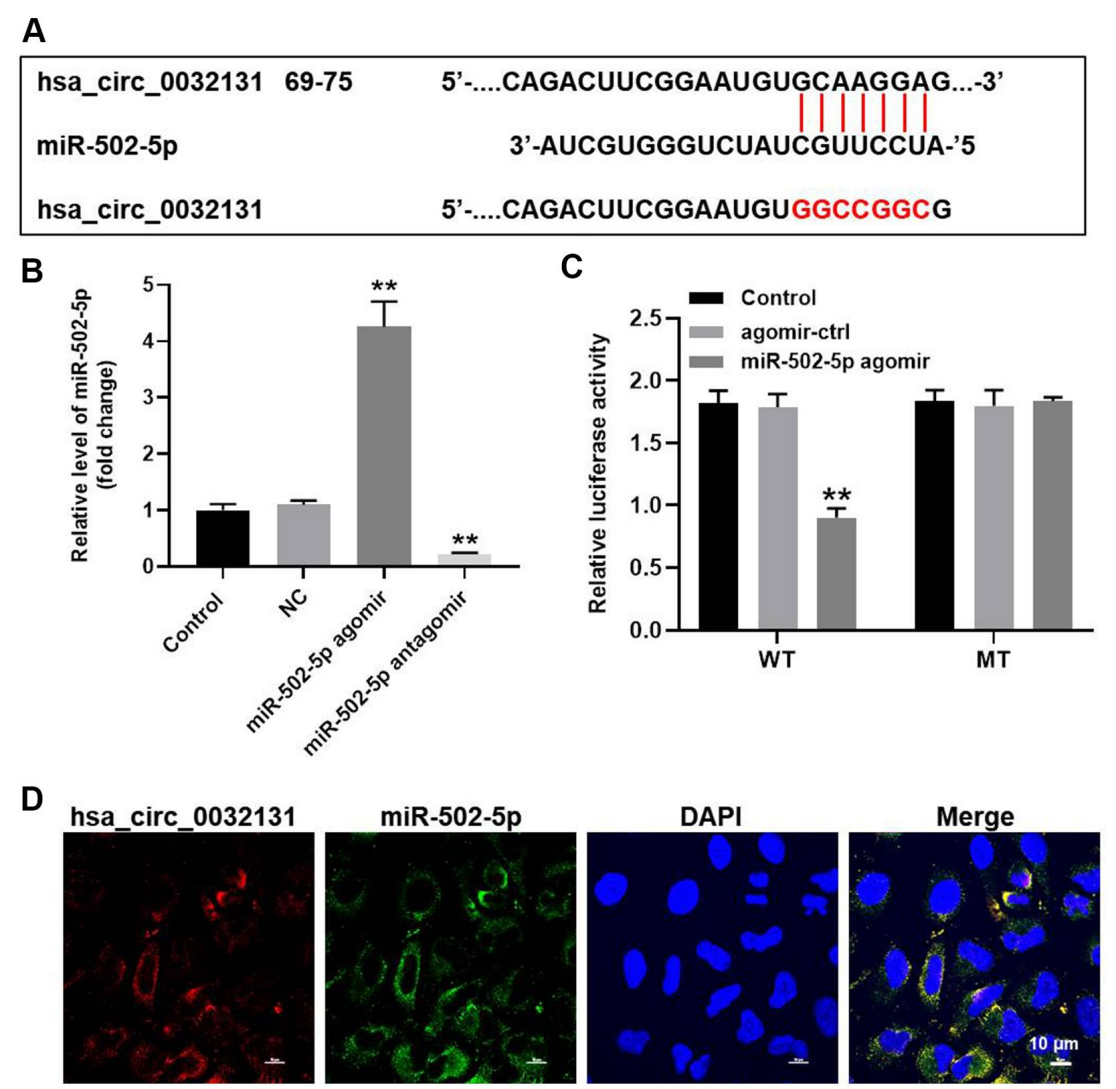
**Hsa_circ_0032131 sponged miR-502-5p in CHON-001 cells.** (**A**) Gene structure of hsa_circ_0032131 at position 69 to 75 indicated the predicted target site of miR-502-5p in its 3'UTR. (**B**) CHON-001 cells were transfected with miR-502-5p agomir/antagomir. Next, the expression of miR-502-5p in CHON-001 cells was measured by RT-qPCR. (**C**) The luciferase activity was measured in CHON-001 cells using the dual-luciferase reporter assay following co-transfection with WT/MT hsa_circ_0032131 3′-UTR plasmid and miR-502-5p. (**D**) The co-localization of hsa_circ_0032131 and miR-502-5p was detected by FISH. ^**^p < 0.01 compared with the control.

To find the miRNAs targeted by hsa_circ_0032131, StarBase and miRDB bio tools were used to study the miRNA–target interactions. Figure 4A reveals a putative target site of miR-502-5p in hsa_circ_0032131. MiR-502-5p level in CHON-001 cells was elevated by miR-502-5p agomir but was reduced by miR-502-5p antagomir (Figure 4B). The luciferase activity of CHON-001 cells transfected with WT-hsa_circ_0032131 and miR-502-5p agomir decreased as compared with the activity of cells transfected with MT-hsa_circ_0032131 (Figure 4C). Furthermore, fluorescence *in situ* hybridization (FISH) showed that miR-502-5p co-localized with hsa_circ_0032131 (Figure 4D). To summarize, hsa_circ_0032131 sponged miR-502-5p in CHON-001 cells.

### Hsa_circ_0032131 shRNA1 inhibited OA progression via the miR-502-5p/PRDX3/Trx1/STAT3 axis

**Figure 5:**
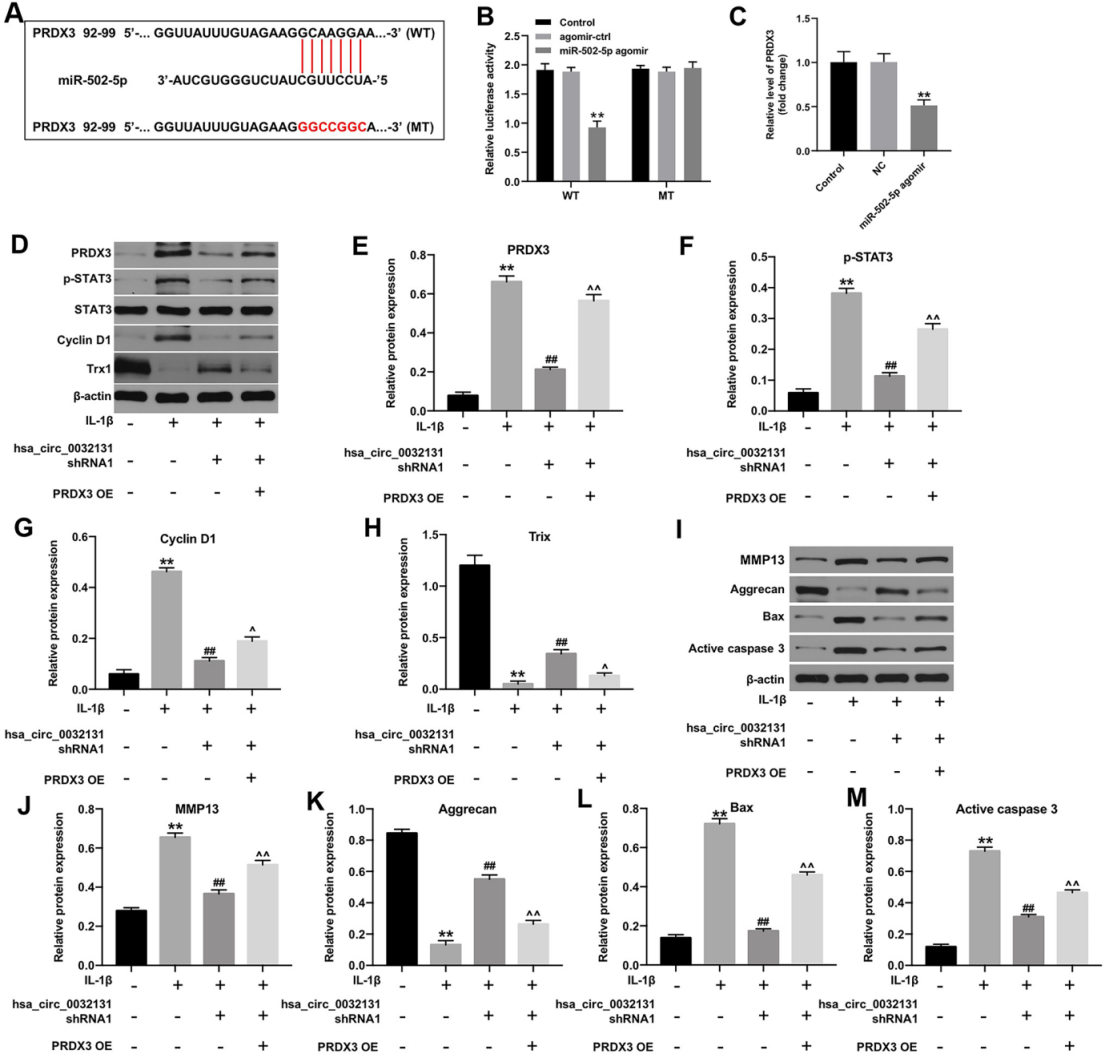
**Hsa_circ_0032131 shRNA1 suppressed OA progression by inhibiting the miR-502-5p/PRDX3/Trx1/STAT3 axis.** (**A**) Gene structure of PRDX3 indicated the predicted target site of miR-502-5p in its 3'UTR. (**B**) The luciferase activity was measured in CHON-001 cells using the dual-luciferase reporter assay following co-transfection with WT/MT PRDX3 3′-UTR plasmid and miR-502-5p. (**C**) The expression of PRDX3 mRNA in CHON-001 cells was detected by RT-qPCR. (**D**) The expression of PRDX3, p-STAT3, STAT3, Cyclin D1, and Trx1 in CHON-001 cells was detected by western blotting. (**E**) The relative expression of PRDX3 was quantified by normalizing it to that of β-actin. (**F**) The relative expression of p-STAT3 was quantified by normalizing it to that of β-actin. (**G**) The relative expression of cyclin D1 was quantified by normalizing it to that of β-actin. (**H**) The relative expression of Trx1 was quantified by normalizing it to that of β-actin. (**I**) The expressions of MMP13, Aggrecan, Bax and active caspase 3 in CHON-001 cells were detected by western blotting. (**J**–**M**) The relative expressions of MMP13, Aggrecan, Bax and active caspase 3 in cells were quantified via normalization to β-actin. ^**^p < 0.01 compared with the control. ^##^*p* < 0.01 compared with IL-1β. ^^^*p* < 0.05, ^^^^*p* < 0.01 compared with IL-1β + hsa_circ_0032131 shRNA1.

TargetScan and miRDB revealed *PRDX3* as a potential downstream target gene of miR-502-5p (Figure 5A). The decreased luciferase activity of CHON-001 cells following transfection with WT-*PRDX3* and miR-502-5p agomir confirmed this finding (Figure 5B). PRDX3 level in was inhibited by miR-502-5p agomir (Figure 5C). Furthermore, hsa_circ_0032131 silencing reduced the expressions of PRDX3, p-STAT3 and cyclin D1, and enhanced Trx-1 level in IL-1β-stimulated cells, while these phenomena were partially reversed by PRDX3 upregulation (Figure 5D–5H). Moreover, hsa_circ_0032131 silencing significantly inactivated MMP13, Bax and active caspase 3, and upregulated the level of aggrecan in IL-1β-induced cells; however, these changes were partially reversed by PRDX3 overexpression (Figure 5I–5M). Altogether, hsa_circ_0032131 shRNA1 alleviated the development of OA by inhibiting the miR-502-5p/PRDX3/Trx1/STAT3 axis.

### PRDX3 overexpression rescued the function of hsa_circ_0032131 shRNA1 in IL-1β-treated CHON-001 cells

**Figure 6:**
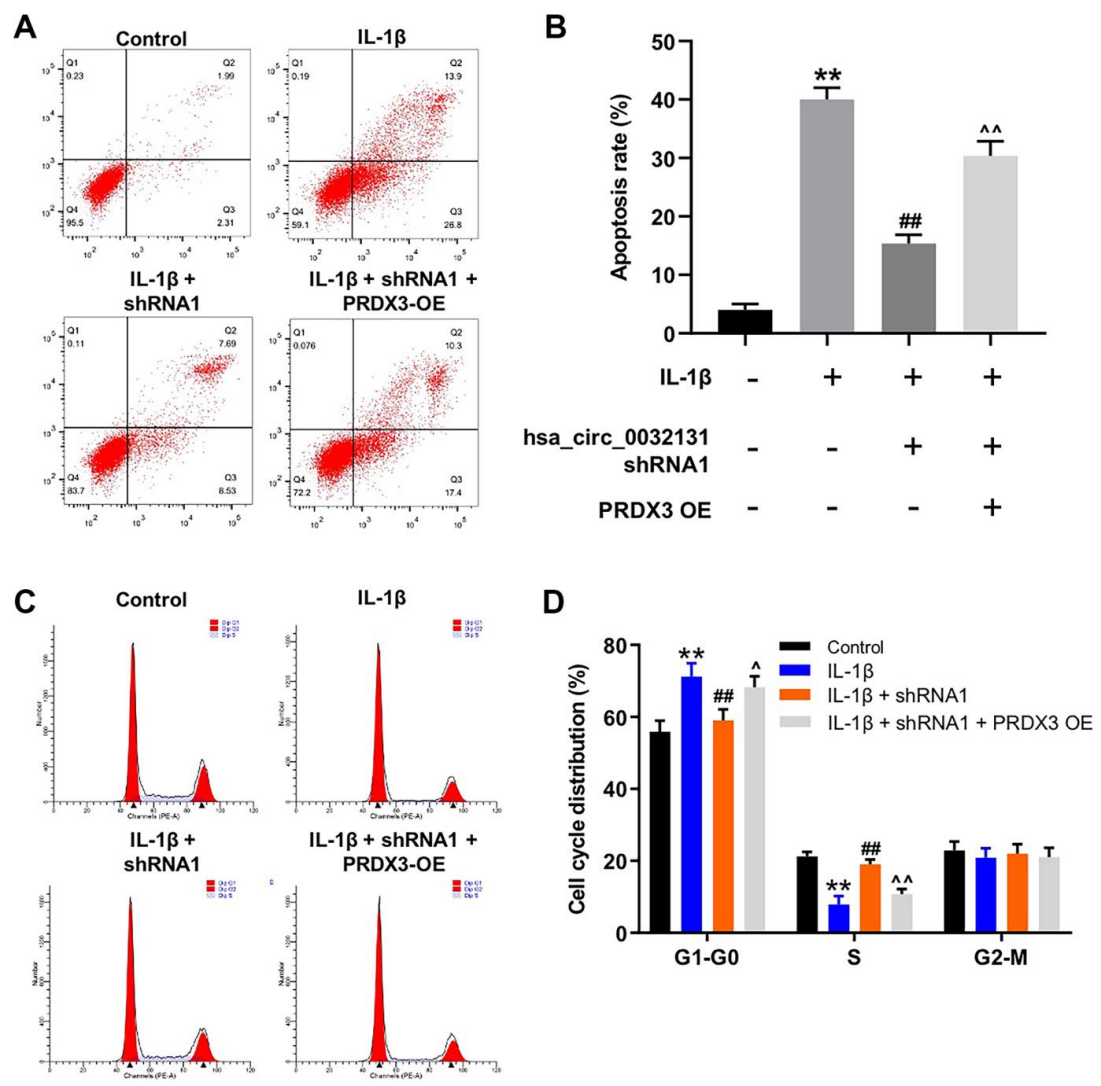
**Overexpression of PRDX3 reversed the effect of hsa_circ_0032131 shRNA1 on the growth of IL-1β-treated CHON-001 cells.** (**A**, **B**) CHON-001 cells were treated with IL-1β, IL-1β + hsa_circ_0032131 shRNA1, or IL-1β + hsa_circ_0032131 shRNA1 + PRDX3 overexpression vector. Next, cell apoptosis was studied by flow cytometry. (**C**, **D**) Cell cycle distribution was studied by flow cytometry. ^**^p < 0.01 compared with the control. ^##^*p* < 0.01 compared with IL-1β. ^^^*p* < 0.05, ^^^^*p* < 0.01 compared with IL-1β + hsa_circ_0032131 shRNA1.

The mechanism by which hsa_circ_0032131 participated in the progression of OA was explored. As indicated by the flow cytometry data shown in Figure 6A, 6B, the anti-apoptotic effect of hsa_circ_0032131 downregulation on IL-1β-stimulated CHON-001 cells was rescued by PRDX3 overexpression. Moreover, IL-1β-induced G1 arrest of CHON-001 cells was rescued by hsa_circ_0032131 silencing, whereas this effect was partially reversed by PRDX3 overexpression (Figure 6C, 6D). In summary, the overexpression of PRDX3 rescued the impacts of hsa_circ_0032131 shRNA1 on the growth of IL-1β-stimulated cells.

### Hsa_circ_0032131 knockdown attenuated OA symptoms *in vivo*

**Figure 7:**
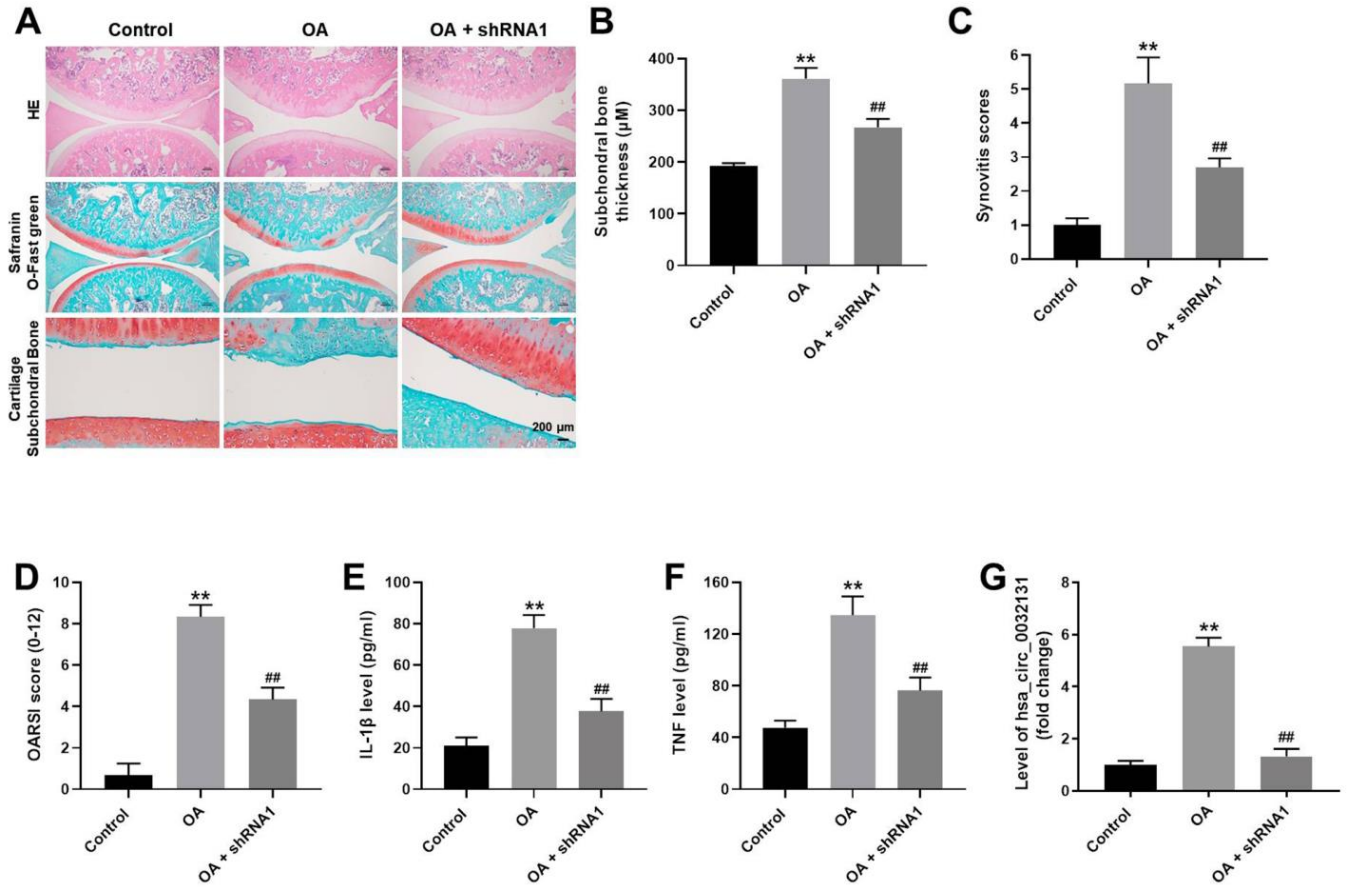
**Knockdown of hsa_circ_0032131 attenuated OA symptoms *in vivo*.** (**A**) Rats were sacrificed and cartilage tissues were collected. Next, OA symptoms in the cartilage tissues were observed by hematoxylin and eosin (H&E) and representative safranin O staining, and by examining the cartilage subchondral bone. (**B**) Subchondral bone thickness was calculated. (**C**) Synovitis scores of cartilage tissues in rats were evaluated. (**D**) The Osteoarthritis Research Society International (OARSI) scores of cartilage tissues were evaluated. The levels of (**E**) IL-1β and (**F**) TNF-α in the plasma of rats were detected by enzyme-linked immunosorbent assay (ELISA). (**G**) The level of hsa_circ_0032131 in rat tissues was measured by RT-qPCR. ^**^p < 0.01 compared with the control. ^##^*p* < 0.01 compared with OA.

To further study the effects of hsa_circ_0032131 on OA, an *in vivo* model of OA was established in rats. As revealed in Figure 7A, cartilage destruction and articular chondrocyte cellularity loss in OA rats were reversed by hsa_circ_0032131 knockdown. The upregulated subchondral bone thickness was observed in OA rats, while hsa_circ_0032131 shRNA1 rescued this phenomenon (Figure 7B). Additionally, hsa_circ_0032131 silencing reduced OARSI scores in OA rats (Figure 7C, 7D). Moreover, the levels of TNF-α and IL-1β in the plasma of OA rats decreased following shRNA1-induced hsa_circ_0032131 silencing (Figure 7E, 7F). Meanwhile, the level of hsa_circ_0032131 was notably increased in OA rats, whereas this effect was reversed following hsa_circ_0032131 knockdown (Figure 7G). Altogether, the knockdown of hsa_circ_0032131 attenuated OA symptoms *in vivo*.

### Hsa_circ_0032131 silencing alleviated OA progression *in vivo* by inhibiting the Trx1/STAT3 signaling

**Figure 8:**
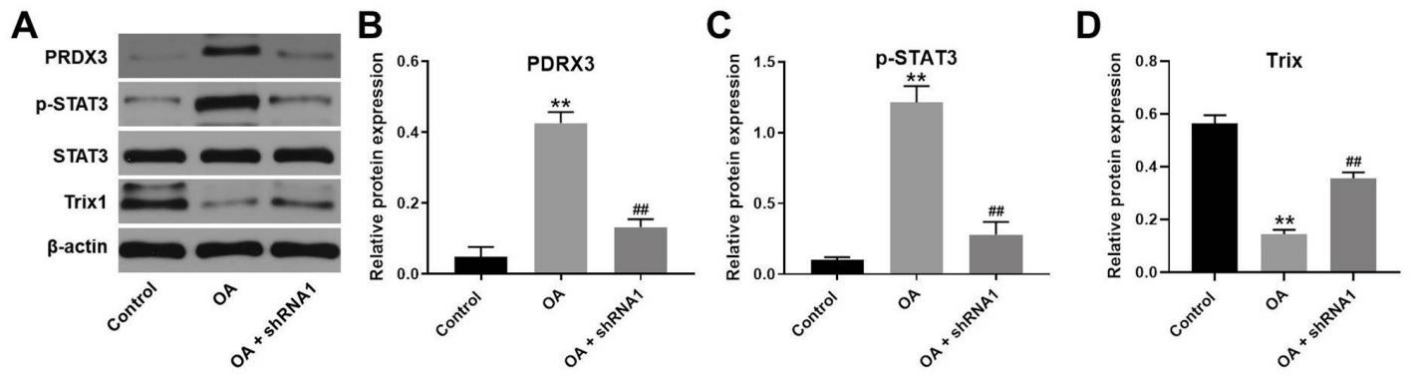
**Silencing of hsa_circ_0032131 alleviated OA progression *in vivo* by inhibiting Trx1/STAT3 signaling.** (**A**) The expression of PRDX3, STAT3, p-STAT3, and Trx1 in rat tissues was detected by western blotting. (**B**) The relative expression of PRDX3 was quantified by normalizing it to that of β-actin. (**C**) The relative expression of p-STAT3 was quantified by normalizing it to that of β-actin. (**D**) The relative expression of Trx1 was quantified by normalizing it to that of β-actin. ^**^p < 0.01 compared with the control. ^##^*p* < 0.01 compared with OA.

We next studied the expression of Trx1 and STAT3 in the tissues of rats by western blotting. The results indicated that protein levels of PRDX3 and p-STAT3 were upregulated in OA rats, whereas this effect was rescued following hsa_circ_0032131 knockdown (Figure 8A–8C). In contrast, Trx1 level in OA rats increased following hsa_circ_0032131 (Figure 8A, 8D). In summary, the silencing of hsa_circ_0032131 alleviated the progression of OA *in vivo* by inhibiting Trx1/STAT3 signaling.

## DISCUSSION

CircRNAs have been implicated in the progression of OA [7, 21]. For instance, circRNA HIPK3 serves as a sponge of miR-124 and contributes to OA pathogenesis [22]. In addition, dysregulation of miRNAs is related with the occurrence of OA [23, 24]. We found that hsa_circ_0032131 functioned as an endogenous competing RNA for miR-502-5p in OA. Moreover, miR-502-5p directly targeted *PRDX3*, a downstream molecule of miR-502-5p, in CHON-001 cells, a finding contrary to that reported by Zhang et al. who found that miR-502-5p inhibited IL-1β-induced chondrocyte injury by targeting *TRAF2* [15]. Nevertheless, because PRDX3 and TRAF2 are intricately associated with OA progression [25, 26], the difference in the results could be attributed to similar functions of these two proteins.

Oxidative stress plays a crucial role in OA development [27, 28]. PRDX3, an antioxidative protein, can regulate cellular redox state [29]. Evidence has shown that antioxidant enzymes, such as dismutase 3 (SOD3), glutathione (GSH), were markedly decreased after OA development [30–32]. However, in this study, PRDX3 expression was significantly increased in IL-1β-treated CHON-001 cells as well as in OA rats, which was inconsistent with previous studies. The reason might be that PRDX3 expression in CHON-001 cells was compensatively elevated by IL-1β. These data indicated that the antioxidant capacity of CHON-001 cells was upregulated during IL-1β, and PRDX3 might play a protective role in IL-1β-treated CHON-001 cells.

Trx1 functions in the tumorigenesis of various cancers and inflammation [33, 34]. We found that the knockdown of hsa_circ_0032131 markedly inactivated PRDX3 and activated Trx1 in IL-1β-induced CHON-001 cells. Jin et al. reported Trx1 as the downstream protein of PRDX3 and found that the expression of PRDX3 was upregulated and Trx-1 level was downregulated in CKI-treated AML cells [35]. In addition, Trx-1 has been shown to exhibit anti-inflammatory and antiapoptotic effects [36]. These data indicated that knockdown of hsa_circ_0032131 attenuated the development of OA by inhibiting the miR-502-5p/PRDX3/Trx1 axis.

The STAT3 pathway participates in the regulation of inflammatory response and progression of OA [37, 38]. Yao et al. indicated that IL-1β significantly upregulated the expression of p-STAT3 as well as induced the apoptosis of chondrocytes [39]. We found that silencing of hsa_circ_0032131 inactivated STAT3 signaling in IL-1β-treated CHON-001 cells. Furthermore, hsa_circ_0032131 shRNA inhibited the expression of Trx1. Lopez-Grueso et al. reported that Trx1 negatively regulated STAT3 signaling [40]. These data indicated that the knockdown of hsa_circ_0032131 alleviated OA symptoms via PRDX3/Trx1/STAT3 signaling.

The present study had certain limitations. First, it focused only on STAT3 signaling. Second, we failed to detect anti-apoptotic proteins. Because other pathways such as the PI3K/Akt signaling are also involved in the development of OA [41], future studies should focus on the effect of hsa_circ_0032131 on these pathways.

In summary, silencing of hsa_circ_0032131 inhibited the progression of OA by suppressing the miR-502-5p/PRDX3/Trx1/STAT3 axis. Thus, hsa_circ_0032131 could act as a new target for OA treatment.

## MATERIALS AND METHODS

### Cell culture

Human chondrocyte cell line (CHON-001) and 293T cells were purchased from ATCC (Manassas, VA, USA) and cultured in RPMI-1640 medium (Thermo Fisher Scientific, Waltham, MA, USA) containing 10% FBS (Thermo Fisher Scientific) and 2 mM glutamine (Sigma-Aldrich, St. Louis, MO, USA) at 37° C. CHON-001 cells were treated with IL-1β (10 ng/mL, Sigma-Aldrich) for 48 h to mimic OA *in vitro* as previously described [42, 43].

### Cell transfection

293T cells (5 × 10^6^ cells/well) were transfected with hsa_circ_0032131 shRNA1, hsa_circ_0032131 shRNA2, PRDX3 overexpression vector (GenePharma, Shanghai, China), or pLVX-IRES-Puro (GenePharma) using the Lipofectamine 3000 reagent. After 48 h of incubation, the lentiviral supernatant was harvested and filtered to obtain viral particles, which were subsequently added to CHON-001 cell suspension (5 × 10^6^ cells/well) and spun at 956×g for 15 min.

In addition, CHON-001 cells were transfected with miR-502-5p agomir, miR-502-5p antagomir, or negative control (NC) RNAs (GenePharma) using Lipofectamine 2000, as previously described 17 [44].

### Reverse transcription-quantitative polymerase chain reaction

TRIzol reagent (Takara, Tokyo, Japan) was applied to isolate total RNA from tissues or cell lines. PrimeScript RT reagent kit (Takara) was applied to reverse transcribe total RNA into cDNA. Then, qPCR was performed using the SYBR Premix Ex Taq II kit (Takara) as follows: 2 min at 94° C, followed by 35 cycles (30 s at 94° C and 45 s at 55° C). The primer sequences used were as follows: hsa_circ_0032131 forward, 5’-GAGATGCTCTGTGGTCACGC-3’ and reverse, 5’-GTCTGCCAGTTTACAGTGACCC-3’; miR-502-5p forward 5’-CACCTGGGCAAGGATTCA-3’ and reverse, 5’-CTCAACTGGTGTCGTGGAGTC-3’; PRDX3 forward, 5’-TCGCAGTCTCAGTGGATTCC-3’ and reverse, 5’-ACAGCACACCGTAGTCTCGG-3’; β-actin forward, 5’-GTCCACCGCAAATGCTTCTA-3’ and reverse, 5’-TGCTGTCACCTTCACCGTTC-3’; and U6: forward, 5’-CTCGCTTCGGCAGCACAT-3’ and reverse 5’-AACGCTTCACGAATTTGCGT-3’. The 2^−ΔΔt^ method was applied for quantification. β-actin or U6 was considered as an internal control.

### Cell counting kit-8 assay

CHON-001 cells (5 × 10^3^ cells/well) were treated with hsa_circ_0032131 shRNA1, IL-1β (10 ng/mL) or IL-1β (10 ng/mL) + hsa_circ_0032131 shRNA1 at 37° C. After that, CHON-001 cells were treated with CCK-8 reagent (10 μL) for 2 h. The absorbance (450 nm) was assessed by a microplate reader.

### Western blotting

RIPA (Beyotime, Shanghai, China) was applied to isolate total protein. BCA (Thermo Fisher Scientific) was used to quantify the total protein. SDS-PAGE (10%) was used to separate proteins (40 μg per lane), and then proteins were transferred onto PVDF membranes (Thermo Fisher Scientific). The membranes were incubated with primary antibodies against aggrecan (Abcam; ab3778, 1:1,000), MMP-1 (Abcam; ab118973, 1:1,000), MMP13 (Abcam; ab219620, 1:1,000), PRDX3 (Abcam; ab136668, 1:1,000), Cyclin D1 (Abcam; ab16502, 1:1,000), STAT3 (Abcam; ab109330, 1:1,000), p-STAT3 (Abcam; ab6503, 1:1,000), and β-actin (Abcam; ab179467, 1:1,000) after blocked with 5% skimmed milk for 1 h. After that, the membranes were incubated with secondary antibodies (HRP-conjugated, Abcam; 1:5,000) for 1 h at room temperature. Protein bands were visualized using the enhanced chemiluminescent (ECL) kit (Thermo Fisher Scientific). β-actin was used for normalization. Image-Pro Plus 6.0 was applied for densitometric analysis.

### Immunofluorescence

CHON-001 cells (5 × 10^4^ cells per well) were seeded overnight. Cells were fixed for 20 min. Then, cells were incubated overnight with anti-Ki67 antibody (1:1,000; Abcam), followed with a goat anti-rabbit IgG secondary antibody (1:5,000; Abcam). The result was observed using a microscope (Olympus, Tokyo, Japan).

### Apoptosis analysis

CHON-001 cells were centrifuged and resuspended in binding buffer. After that, 5 μL Annexin V-fluorescein isothiocyanate (FITC) and PI were added to the cells at 4° C for 15 min. The cell apoptosis was analyzed by flow cytometry (Becton, Dickinson and Company, Franklin Lake, NJ, USA).

### Prediction of downstream targets

The target gene of hsa_circ_0032131 was predicted using a publicly available program (StarBase, http://starbase.sysu.edu.cn/). TargetScan and miRDB were used to predict the target of miR-502-5p.

### Dual-luciferase reporter assay

The partial sequences of hsa_circ_0032131 and the *PRDX3* 3’- UTR containing the sites of miR-502-5p were synthesized by GenePharma. The aforementioned sequences were cloned into the pmirGLO vectors (Promega, Madison, WI, USA) for establishment of wild-type (WT) or mutant (WT) reporter hsa_circ_0032131 and *PRDX3* vectors. The WT or MUT miR4435-2HG vector was transfected into cells along with miR-502-5p agomir using Lipofectamine 2000 reagent. The data were quantified and normalized to Renilla luciferase activity.

### Fluorescence *in situ* hybridization

The co-localization of miR-502-5p and hsa_circ_0032131 in the cytoplasm was detected using FISH, as described previously [45].

### *In vivo* model of OA

Fifteen Wistar rats (12-week-old) were obtained from the Chinese Academy of Sciences (Shanghai, China). Rats were injected with saline (control and OA group, 200 μL) or hsa_circ_0032131 shRNA1 (OA + hsa_circ_0032131 shRNA1; 2 × 10^7^ plaque-forming units (PFUs), 200 μL, twice a week) via joint cavity. To induce OA *in vivo*, Rats in OA or OA + hsa_circ_0032131 shRNA1 group were treated with medial meniscus (DMM) surgery [46]. To mimic OA *in vivo*, rats were intraperitoneally injected with 40 mg/kg pentobarbital (2%), and then the joint capsule of the right knee was incised medially, as described previously [46]. Afterward, microsurgical scissors were used to transect the medial meniscotibial ligament [46]. Rats were sacrificed at the end of the study for collection of plasma and knee joint tissues. The protocols for animal care and use of laboratory animals were approved by Tianjin Hospital (No. 20200221).

### Histopathological analysis

Safranin O and Fast Green was used to stain tissue specimens. The morphology of the subchondral bone and cartilage was observed under a microscope. The tibial plateau and medial femoral condyle were evaluated by OARSI scoring system [47]. In addition, hematoxylin and eosin (H&E) staining was performed to investigate the status of cartilage destruction.

### Enzyme-linked immunosorbent assay

The levels of IL-1β and TNF-α in the plasma of rats were assessed using an ELISA kit (MultiSciences Lianke Biotech Co., Ltd, Hangzhou, China).

### Statistical analysis

Data are presented as the mean ± SD. One-way analysis of variance and Tukey’s post hoc tests were used for comparisons between ≥3 groups. Student’s t-test was used for comparisons between tumor tissues and adjacent normal tissues of the same patients, while an unpaired Student’s t-test was used for comparisons between unpaired groups. P<0.05 was considered to indicate a statistically significant difference.

### Ethics approval statement

This study was approved by the Ethics Committee of Tianjin Hospital (No. 20200221).
